# The timing of binding and segregation of two compound aftereffects

**DOI:** 10.1016/j.visres.2011.02.017

**Published:** 2011-05-11

**Authors:** David P. McGovern, Sarah Hancock, Jonathan W. Peirce

**Affiliations:** Nottingham Visual Neuroscience, School of Psychology, University of Nottingham, Nottingham, UK

**Keywords:** Contours, Plaids, Adaptation, Binding, Segregation

## Abstract

Temporal information in a scene is thought to be an important cue for visual grouping of local image features into a single object. The majority of studies on this topic have attempted to determine the conditions that facilitate segregation of a figure from a cluttered background. Here we examine the temporal characteristics of two aftereffects that appear to have roles in visual integration: the curvature aftereffect (CAE; Hancock & Peirce, 2008) and plaid-selective contrast adaptation (Peirce & Taylor, 2006). Both aftereffects used a “compound adaptation” paradigm measuring adaptation to a compound stimulus that cannot be explained by adaptation to its components presented in isolation. The temporal tuning characteristics of the two aftereffects differed in three distinct ways. First, plaid-selective adaptation was very sensitive to temporal phase asynchronies, while the CAE was not. Second, while both aftereffects showed integration of alternating components above 4 Hz, for plaids the overall magnitude of adaptation was less than to synchronous stimuli and was eliminated at the highest frequencies. Finally, plaid-selective adaptation demonstrated a low-pass dependency for temporal flicker frequency of synchronous gratings, whereas the CAE did not. Overall, these results suggest that at least two different mechanisms are involved in the binding/segregation of local signals into compound patterns: one with high temporal resolution that allows rapid parsing of plaid patterns into their components and one with a coarser temporal sensitivity that mediates the CAE.

## Highlights

► Plaid and curvature adaptation effects show different dependencies for temporal phase and frequency. ► Plaid adaptation is highly sensitive to the temporal phase of its constituent gratings. ► Our results suggest that at least two neural mechanisms underlie the combination of local features into compound patterns.

## 1 Introduction

Analysis of a visual scene requires the solution of two related problems; combining the sensory information that belongs to one object (binding) and dissociating that information that comes from separate objects (segregation). These problems became a major focus of the Gestalt psychologists who revealed a number of principles that enable the perceptual grouping of similar features. Similarities of spatial features such as orientation, luminance, and colour were deemed to provide potent cues to aid grouping, while dissimilarity in these attributes leads to segregation (Kanizsa, 1979; Koffka, 1935). ‘Common fate’, the tendency of the visual system to combine objects that move together into a single perceptual unit, also results in perceptual grouping. Since these initial reflections, much research has focused on how other fluctuations of temporal information (e.g. differences in luminance over time and temporal phase) might influence grouping.

Many of these studies have aimed to determine whether temporal asynchrony can act as a cue for figure-ground segregation. In some studies, a temporal phase lag of as little as 5–16 ms between two sets of flickering elements can facilitate, or even be sufficient for observers to segment a figure from its background (Fahle, 1993; Forte, Hogben, & Ross, 1999; Kojima, 1998; Leonards, Singer, & Fahle, 1996; Rogers-Ramachandran & Ramachandran, 1991, 1998; Usher & Donnelly, 1998). In an extreme example, Fahle (1993) reported figure-ground segregation in arrays of flickering dots based purely on flicker phase lags of as little as 6–7 ms between target and background dots, while Hancock, Walton, Mitchell, Plenderleith, and Phillips (2008) found that slightly longer asynchronies of 20–40 ms were effective for path segregation in Gabor arrays.

Aspects of temporal structure that can be used for figure-ground segregation are not limited to onset or phase asynchronies. Temporal synchrony of changes in stimulus characteristics such as contrast or orientation also acts as a major signal for grouping (Alais, Blake, & Lee, 1998; Kandil & Fahle, 2001; Sekuler & Bennett, 2001). Lee and Blake (1999a) studied Gabor element arrays where the direction of motion of each element reversed direction irregularly over time. Figure and ground elements had different patterns of reversals (termed ‘point processes’) but did not differ in any other way. Under these conditions the figure was easily detected with displays of only a few hundred milliseconds. Their results may be explained by low-pass temporal filtering, without requiring the existence of a mechanism sensitive to the fine temporal structure (Adelson & Farid, 1999), although subsequent experiments aimed to rule out such an explanation (Lee & Blake, 1999b).

Temporal structure does not always contribute to spatial grouping, however. Kiper, Gegnfurtner, and Movshon (1996) measured observer performance in discriminating the orientation of a rectangular test patch against a background of oriented line elements. In this experiment both spatial similarity (difference in the orientation of line segments) and temporal similarity (duration of a temporal phase lag) were varied independently to determine the exact role of each cue. Temporal phase differences had little influence on texture segmentation; observers appeared to use only the more salient spatial differences between target and surround to make their judgment. Similarly, small spatial distortions of a bi-stable stimulus can significantly bias the observer’s dominant percept, whereas temporal cues are virtually ignored (Fahle & Koch, 1995). These seemingly contradictory findings may be the result of interactions with strong spatial cues. For example, Leonards et al. (1996) found that figure-ground could be defined purely by temporal phase, but if salient spatial cues were present they dominated over temporal cues. Furthermore, non-informative temporal information in the array did not disrupt binding based on spatial information. Similarly, Usher and Donnelly (1998) showed that temporal phase cues could bias grouping in an otherwise ambiguous display, and could facilitate detection of a collinear target or define a randomly oriented target in a line segment array, but the efficacy of these cues was constrained by the presence of spatial cues.

Under natural viewing conditions, temporal structure may be noisy and unpredictable, due to object motion, eye movements and blinks, or changes in illumination (amongst other things). Thus, the visual system needs to be able to deal with interrupted visual input. When parts of the same stimulus are presented at different times, binding appears to be possible over relatively long durations, suggesting an integration window during which separate stimuli may be grouped together. Estimates of the duration of this window have varied. Leonards et al. (1996) reported that binding could occur across temporal intervals of up to 25 ms in a figure-ground task, while Altmann, Eckhorn, and Singer (1986) suggest that temporal dispersion could be closer to 150 ms without disrupting binding of spatially separated features. Other studies have considered the interval over which stimuli appear to be simultaneous, reporting that stimuli are seen as simultaneous if they occur within around 50 ms of each other (Elliott, Shi, & Kelly, 2006; VanRullen & Koch, 2003).

Here, we use a novel approach to investigate the role of temporal structure in the grouping of simple grating elements into conjunctions. The method involves simultaneous adaptation to a compound stimulus comprising two gratings, either as a plaid or a chevron-like contour, in one location and the two component gratings presented in alternation in a different location. By presenting compound test probes in both locations we can determine adaptation effects that are specific to the compound stimulus over and above adaptation to its parts. Using this *compound adaptation* technique (Peirce & Taylor, 2006) we vary the temporal parameters of the adapting stimuli and examine the conditions under which the component gratings comprising plaids and contours are grouped (compound adaptation occurs) or segregated (compound adaptation is disrupted). Two aftereffects are compared; the curvature aftereffect (CAE) and plaid-selective contrast adaptation, both of which have been discussed previously (Hancock & Peirce, 2008; McGovern & Peirce, 2010). For both effects, when the spatial frequency differs strongly between the components, causing them to be seen less as a coherent single pattern, the adaptation effect was reduced (Hancock, McGovern, & Peirce, 2010), indicating a potential role for the underlying mechanisms in perceptual grouping or binding. This similarity between the tuning profiles of each aftereffect for spatial frequency suggested that they both might arise from a common binding mechanism. However, previous work demonstrates that the spatial proximity of stimulus elements influences how temporal information in the stimulus is used for grouping processes (Holcombe & Cavanagh, 2001). Thus, measuring the temporal dependencies of both aftereffects might help elucidate whether the aftereffects arise from one general binding mechanism or two distinct ones.

We find that the two different aftereffects differ in their sensitivity to temporal structure. There was an abrupt decrease in plaid-selective adaptation with very small differences (50 ms) in the temporal phase of the plaid components. This was not the case for the CAE, which required much larger asynchronies of the components to significantly affect the magnitude of the adaptation effect. Both aftereffects showed integration of alternating components above 4 Hz, but for plaids, adaptation was less than to synchronous stimuli and was eliminated at the highest frequencies. Furthermore, plaid-selective adaptation showed low-pass tuning for temporal frequency of synchronous gratings, whereas the CAE peaked at intermediate frequencies.

## 2 General methods

All the experiments in this paper used a ‘compound adaptation’ paradigm, designed to measure adaptation to a compound stimulus beyond that predicted by adaptation to its constituent parts. The method aims to study the tuning of mechanisms that respond selectively to the presentation of such conjunctions. Two patches on opposite sides of the visual field are adapted simultaneously – one to a stimulus consisting of two gratings presented together (the compound field), and one to the same two grating stimuli presented in isolation, and alternating (the component field). The patches are equated for the duration and intensity of presentation of the components in the two fields. A test stimulus is then presented in both adapted locations and the point of subjective equality is determined. As presentation of the component gratings was equated in the adapting locations any aftereffect due to adaptation to the components alone should be equal on both sides. Therefore, any *difference* in the adaptation effect between the two sides must be due to adaptation to the compound as a whole. One point to note here is that cross-orientation suppression (Morrone, Burr, & Maffei, 1982) cannot account for the compound adaptation effect. Such inhibition would lead to the plaid being reduced in contrast leading to a smaller plaid adaptation effect, where critically we find greater adaptation to the plaid. In this sense, any compound adaptation effect is only made apparent after overcoming the effects of cross-orientation suppression.

**Fig. 1:**
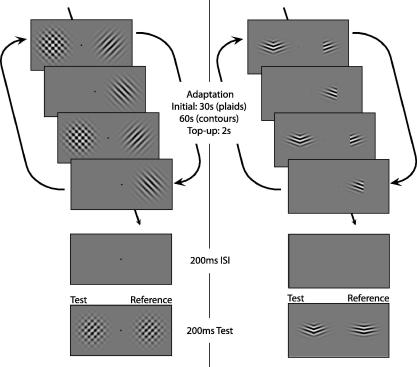
Schematic of the standard procedure for plaid-selective adaptation (left) and the CAE (right). In the component field (right), individual gratings alternated every second, and in the compound field (left), the compound stimulus alternated with a blank field. Note that for convenience the component and compound stimuli are depicted as alternating at the same times. In the actual experiment, the relative temporal phase of the alternations in the two hemi-fields was given a random offset that differed every trial to avoid synchronous alternations in the two hemi-fields. For this experiment the reference probe angle was fixed and the test probe angle (compound side) was varied according to a staircase procedure to find the PSE. The probe stimuli consisted of compound stimuli. For the plaid aftereffect the test probe varied in contrast and observers judged which probe had the higher contrast. For the CAE the test probe varied in curvature and on each trial observers judged which probe stimulus appeared to have the smaller angle. Each observer performed a minimum of four staircases for each adapting configuration. Different temporal sequences were used for the compound adaptor in each experiment (see Section 2 for details).

Two different forms of compound adaptation were examined (see Fig. 1). In the first, plaid-selective adaptation, the compound stimulus was comprised from two fully overlapping gratings each at half contrast, giving rise to a full contrast plaid. Adaptation to this stimulus results in a greater decrease in the apparent contrast of a subsequently presented test probe than the decrease in the equivalent component stimuli. In the second, the curvature aftereffect (CAE), the compound stimulus was comprised of two gratings that are presented adjacent to each other to form a chevron-like contour. Adaptation to this stimulus leads to a straight test contour appearing more curved in the opposite direction, an effect that cannot be solely explained by local tilt aftereffects (Hancock & Peirce, 2008).

**Fig. 2:**
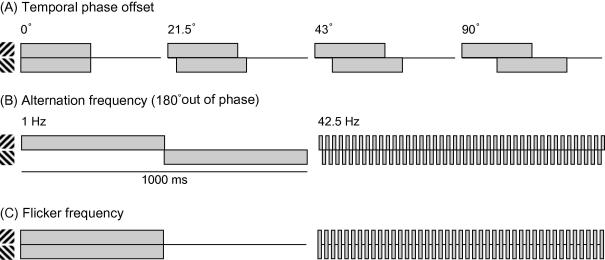
Schematic presentation of the stimulus sequences within the compound adaptor in each experiment. In all cases the component adaptor comprised the component gratings alternating at 1 Hz, except Experiment 1 plaid adaptation where the components alternated at 0.5 Hz. (A) Experiment 1. Components were presented alternating with a blank screen with a frequency of 1 Hz (contours adaptation) or 0.5 Hz (plaid adaptation), but an offset was introduced so that the gratings either appeared synchronous (shown in A), alternating (shown in B) or with a phase asynchrony of 21.5–90° (59–247 ms) for contours or 19–144° (47–800 ms) for plaids. (B) Experiment 2. The component gratings comprising the compound stimulus alternated at one of seven frequencies between 1 Hz and 42.5 Hz. (C) Experiment 3. The component gratings were presented simultaneously alternating with a blank screen at one of seven frequencies between 1 Hz and 42.5 Hz.

Three different temporal manipulations of the compound stimulus were examined for each aftereffect (see Fig. 2). In Experiment 1, the components comprising the compound stimulus were presented with a varying temporal phase offset to examine how the underlying mechanisms represent conjunctions across time and how this affects adaptation. In Experiment 2, the components were presented completely out of phase (alternating) and the alternation rate was varied to examine integration across temporally separate events. Finally, in Experiment 3, the components were always presented in synchrony and the temporal frequency was varied to determine whether the neural mechanisms responsible for the aforementioned aftereffects integrate over separate stimulus presentations (i.e. across different stimulus cycles).

### 2.1 Participants

Participants consisted of six healthy volunteers (two experienced observers and four naïve participants) with normal or corrected-to-normal vision, who gave their consent. Of these, experienced observers SH and DM took part in all Experiments, LS participated in all the contour adaptation experiments, CS participated in the plaid condition for Experiment 1, and SQ participated in the plaid condition for Experiment 3. All procedures were approved by the School of Psychology Ethics Committee, University of Nottingham, UK.

### 2.2 Apparatus

For the contour adaptation experiments, stimuli were presented on a computer-controlled cathode-ray-tube (CRT) monitor (Vision Master Pro 513, liyama) at a resolution of 1024 × 768 pixels and at a refresh rate of 85 Hz with a mean luminance of 49.56 cd/m^2^. The observer’s head was stabilized in a chin-rest 52 cm from the monitor with the viewable area subtending 43.6° visual angle. For the plaid adaptation experiments, stimuli were presented on a computer-controlled cathode-ray-tube (CRT) monitor (Vision Master Pro 454, liyama) at a resolution of 1152 × 864 pixels and at a refresh rate of 85 Hz with a mean luminance of 108.3 cd/m^2^. The viewing distance was 57 cm from the monitor giving a viewable area of 40.5° of visual angle.

Both monitors were driven by 14-bit digital-to-analogue converters (Bits++, Cambridge Research Systems, Cambridge, UK). Stimuli were presented and data collected using the PsychoPy stimulus generation library (Peirce, 2007a, 2007b). They were calibrated using a photo-spectrometer (PR650, Photo Research, Chatsworth, CA, USA) to gamma-correct the red, green and blue (RGB) guns independently and the gamma correction was verified psychophysically using a 2nd-order motion-nulling procedure (Ledgeway & Smith, 1994).

### 2.3 Plaid adaptation

#### 2.3.1 Stimuli

Stimulus parameters were as used for the plaid-selective aftereffect in Hancock et al. (2010). Plaids were constructed from the linear combination of two luminance-modulated sinusoidal gratings at oblique angles, ±45° from vertical. Each grating contributed equal contrast to the plaid (50% of maximum contrast of 0.98 Michelson). All stimuli were presented in a Gaussian envelope with a standard deviation of 1.33° visual angle (such that the stimulus had a diameter of 8° at the point where it fell below 1% contrast). The spatial frequency of the gratings was 0.8 c/°. The spatial phase of the stimulus was randomly jittered every 200 ms to prevent retinal afterimages. Component gratings were centred at 6° visual angle either side of the fovea on the horizontal meridian.

#### 2.3.2 Procedure

The procedure for plaid adaptation is shown schematically in Fig. 1 (left panel). Participants were adapted to a pair of component gratings at different locations on the retina. During adaptation both gratings were presented simultaneously as a full contrast plaid in one visual hemi-field (compound field) and individually as two half-contrast gratings, alternating every second, in the other hemi-field (component field). The plaid stimulus alternated with a blank field every second to equate the exposure time for each of the two gratings in both hemi-fields. The temporal phases of the alternations in each hemi-field were independently randomized. After adaptation, participants compared the contrast of a plaid probe at the same retinal location that it had itself been adapted (the test probe) with one in the opposite location (the reference probe) and were required to report which side had the higher apparent contrast. The reference probe took a fixed contrast value of 0.42, while the contrast of the test probe gradually decreased or increased in steps using an adaptive 1-up, 1-down staircase procedure designed to maintain stimulus presentation near the point of subjective equality (PSE). Each staircase consisted of 50 test presentations of the probe stimuli.

The initial period of adaptation lasted for 30 s and was ‘topped-up’ with another 2 s of adaptation prior to each trial. This was followed by a 200 ms inter-stimulus interval (ISI) before presentation of the probe stimuli for 200 ms. A fixation spot was visible for the entire trial. Observers pressed one of two keys to make a 2AFC response indicating the side on which the stimulus appeared to have higher contrast, triggering the next trial to commence with another ‘top-up’ adaptation period.

Observers were adapted in separate sessions to trials with the compound adaptor on both left and right sides of fixation in order to control for any internal side-biases (that is, a tendency for the observer to have stronger adaptation in one visual hemi-field than the other, or to press one key more often than the other). Each observer collected a minimum of four staircases (a block) for each stimulus condition on each side of fixation. To prevent crossover adaptation between conditions, a minimum time of 1 h (and typically much longer) was left between blocks.

### 2.4 Contour adaptation

#### 2.4.1 Stimuli

Contour stimuli were constructed from two luminance-modulated sinusoidal gratings oriented to form an oblique, V-shaped contour of 140°. In a previous paper (Hancock & Peirce, 2008) two partially-overlapping Gabor patches were used to create a continuous contour. Using that method, the range of orientations that can be presented without creating an artifact in the centre of the stimulus is limited. To avoid this problem, the stimuli in this study abutted the component gratings along a hard edge and then the entire stimulus was masked by a Gaussian profile, a configuration used by Hancock et al. (2010). When combined, these components create continuous contours with a more flexible degree of curvature. Together the two components form an ellipse in a Gaussian envelope with a standard deviation of 0.83° visual angle in the vertical direction and 1.67° in the horizontal direction such that the stimulus had a width of 10° visual angle and a height of 5° at the point where it fell below 1% contrast. All stimulus parameters were as in Hancock et al. (2010) for the CAE. The stimuli were always presented at maximum contrast (0.98 Michelson). The spatial frequency of the gratings was 1.1 c/°. Component gratings were positioned so that the centre of the contour (the point at which the two components abutted) was centred at 6.5° visual angle either side of the fovea on the horizontal meridian. The spatial phase of the stimulus was randomly jittered every 200 ms to prevent retinal afterimages.

#### 2.4.2 Procedure

The procedure for contour adaptation was very similar to the plaid adaptation procedure described above and is shown schematically in Fig. 1 (right panel). During adaptation both gratings were presented simultaneously as a contour in one visual hemi-field (compound field) and individually as two component gratings, alternating every second, in the other hemi-field (component field). The initial adaptation period was 60 s. Otherwise all timings were the same as in the plaid experiments. Observers were required to report the side on which the probe stimulus appeared to have the greater curvature (more acute angle) in a 2AFC task. The repulsive nature of the tilt aftereffect results in reduced apparent curvature of both probes and any additional ‘curvature aftereffect’ (CAE) would result in a further reduction. The staircase increased or decreased the contour angle of the test probe to home in on the point at which observers perceived the two probe stimuli to have equal curvature (PSE).

As in the plaid adaptation, a minimum of four staircases with the compound adaptor in the left hemi-field and four staircases with the compound adaptor in the right hemi-field were collected for each stimulus condition.

### 2.5 Data analysis

**Fig. 3:**
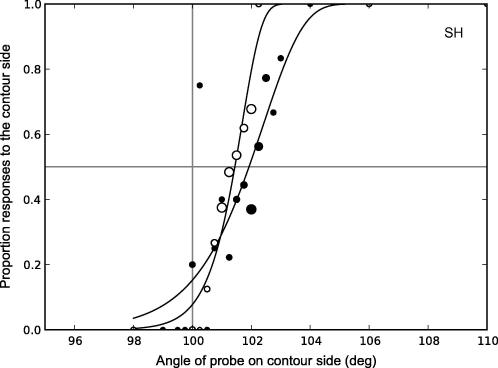
Sample psychometric functions for compound stimuli on the left (filled symbols) and right (open symbols) of fixation (observer SH, contour adaptation, 1 Hz, synchronous components). Symbol size indicates the relative number of responses underlying each data point (actual numbers vary from 1 to 46 responses per point). The horizontal grey line indicates the point of 50% probability of responding on the compound side. The vertical grey line (100° on *x* axis) represents the point of veridical equality. The PSE is calculated from the average of the points where the left and right side functions cross the 50% probability line.

Each participant collected at least 200 trials for each condition with the compound adaptor on each side of fixation (4 × 50-trial staircases). The responses for each probe stimulus intensity level (either contrast difference or angular difference) were averaged for each observer and Weibull functions were fit to the averaged data. The PSE was derived from this fit as the point at which the observer was at 50% probability of responding on the compound side. Using this method, all data contribute to the calculation of the PSE, rather than only data from those trials on which reversals occur, and a full psychometric function can be recovered. It should be noted that data points near the PSE have more trials contributing to each point, as a result of the staircase procedure itself. Fig. 3 shows an example pair of psychometric curves.

For each condition, we quantified the magnitude of compound adaptation as the amount of additional contrast (plaid experiments) or curvature (contour experiments) required in the compound adapted hemi-field for the probes to appear equal. This is the mean shift in the PSE from the point of veridical equality. These average PSE values (selective adaptation) are plotted as differential effects. That is, selective adaptation effects are plotted as adaptation to the compound above and beyond adaptation to the components.

Additionally, plaid adaptation effects are expressed in decibels using the following equation:

$$\mathrm{db}=20\;*\;\text{log}10(C_{\text{adapt}}/C)$$

where *C* is the contrast of the reference probe and *C*_adapt_ is the Michelson contrast value required to equate the test probe to the reference.

Functions were fit with 5000 within-subject bootstrap re-samples for each condition (each with 200 trials for each side as in the original dataset) so that, for each re-sample, a whole new pair of psychometric functions could be derived (one for each side on which the compound was adapted). The PSE values for each pair of functions were averaged to account for side bias then used to derive 95% confidence intervals of the PSE for each observer in each condition.

## 3 Experiment 1. The effect of component asynchrony

To investigate whether the mechanisms involved in the binding of grating components into conjunctions are sensitive to the temporal synchrony of their components, we examined plaid-selective adaptation and the curvature aftereffect (CAE) produced when the components comprising the ‘compound’ adaptor were presented with a temporal phase offset. By presenting the constituent gratings in an asynchronous manner the coherent perception of the compound adaptor should, at some point, be broken. The key question here is *how* this temporal phase lag between the components affects compound adaptation. In the extreme case, when the components stimuli are in temporal anti-phase (data presented in Experiment 2), the compound and component side adaptors are identical so we should see no effect of compound adaptation. Depending on the temporal sensitivity of the underlying conjunction mechanism, partial temporal phase offsets could have one of two different effects on the magnitude of compound adaptation. If the underlying mechanisms utilise temporal cues to group the components we might expect an abrupt decrease in adaptation with a temporal offset of the components. That is, compound adaptation would greatly diminish at the point where the perceived coherence of the adaptor is broken, with further temporal phase lags leading to relatively small drops in adaptation. However, if the underlying mechanisms ignore temporal phase cues as an aid for grouping, we might expect to see the magnitude of adaptation decrease as a function of the amount of time that the components appear together as a compound, resulting in a more gradual decrease in adaptation with increasing offset.

Fig. 2A provides samples of the stimulus presentation sequence for the components comprising the compound adaptor in terms of the temporal phase. Exact timings differ for the plaid and contour adaptation experiments.

### 3.1 Plaid adaptation

For plaid adaptation, the component gratings in both the compound and component adaptors were presented with a frequency of 0.5 Hz, i.e. each grating alternated with a blank field every second. On the component side the two gratings were always presented in alternation. On the compound adaptor side the two gratings comprising the plaid adaptor were presented with a temporal phase asynchrony of 0°, 19°, 36°, 72°, or 144° of phase angle (0, 106 ms, 200 ms, 400 ms, or 800 ms; exact timings dependent on the frame rate of the monitor). Observers DM and SH also completed an additional condition with an 8.5° phase offset (47 ms) asynchrony.

**Fig. 4:**
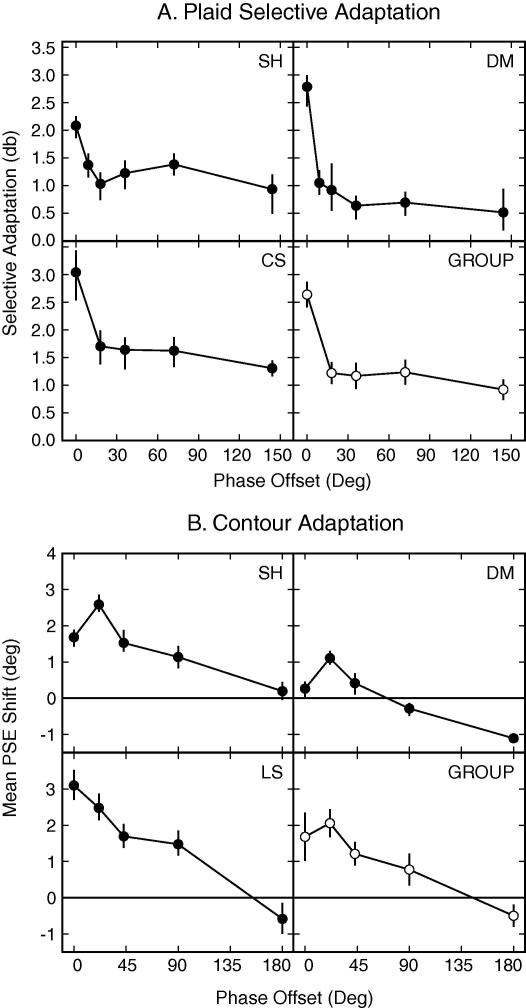
Magnitude of (A) the plaid adaptation effect (mean PSE shift in decibels) and (B) the CAE (mean PSE shift in degrees), at different component temporal phase offsets (Experiment 1) for individual observers and across the group. Error bars for individual data indicate 95% confidence intervals. Error bars for group data indicate the SEM across observers (in each case *N* = 3). 0 on the *y*-axis indicates the point of veridical equality.

**Fig. 5:**
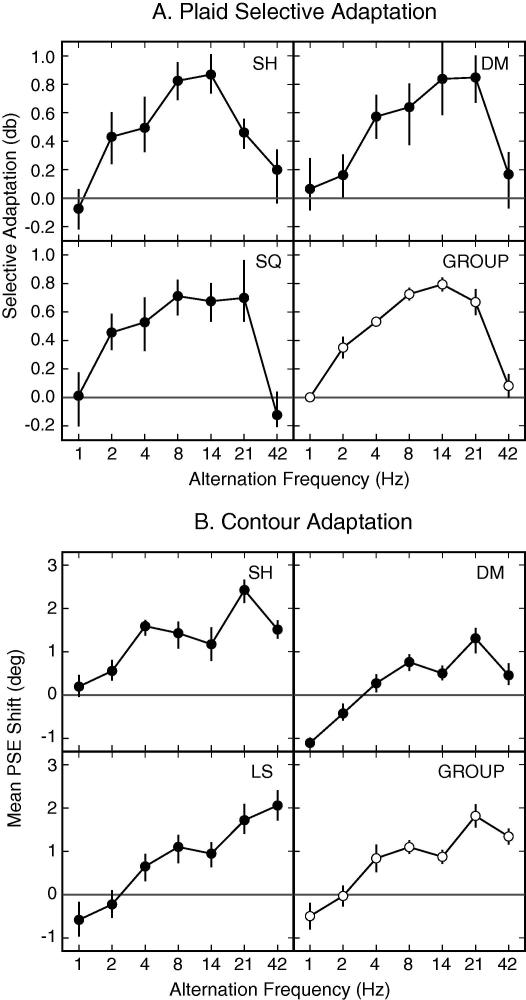
Magnitude of (A) the plaid adaptation effect (mean PSE shift in decibels) and (B) the CAE (mean PSE shift in degrees), at different temporal frequencies (Experiment 2) for individual observers and across the group. Error bars for individual data indicate 95% confidence intervals. Error bars for group data indicate the SEM across observers (in each case *N* = 3). 0 on the *y*-axis indicates the point of veridical equality.

Mean PSE values for all asynchronies for all three observers, individually and across the group, are shown in Fig. 4A as a function of phase angle. When the components were presented synchronously the mean adaptation effect across observers was 2.59 db. This reflects a greater reduction in the perceived contrast of the test plaid presented in the compound field than the reference probe presented in the component field. When an asynchrony was introduced between the two components there was an abrupt decrease in the magnitude of compound adaptation. This reduction in adaptation at the smallest offset tested was statistically significant (95% confidence intervals measured using a bootstrap analysis did not overlap) for all observers. Much smaller decreases in plaid-selective adaptation were observed with further temporal phase lags and a small compound adaptation effect remained for all conditions. Data when the component gratings comprising the plaid were completely out of phase (i.e. alternating) were collected as part of Experiment 2 and as expected no significant plaid-selective aftereffect was found (see Fig. 5A, 1 Hz condition). It is unclear why a residual adaptation effect is observed in the conditions between the initial decrease in plaid-selective adaptation and when the components are completely out of phase. One reason may be that two separate mechanisms (one phase sensitive, the other not) contribute to the plaid-selective adaptation effect, a possibility that we consider in Section 6.

As mentioned previously in Section 2, cross-orientation suppression (Morrone et al., 1982) cannot account for the sharp decrease in plaid adaptation with small temporal phase differences; with the increased offset between the gratings comprising the plaid, the degree of lateral inhibition between detectors for the component gratings should be decreased, resulting in greater effective contrast of the compound adaptor and an increased adaptation effect (see McGovern & Peirce, 2010).

### 3.2 Contour adaptation

Stimulus presentation sequences for the contour adaptation procedure are very similar to those for the plaid adaptation experiment. The component gratings in both the compound and component adaptors were presented with a temporal frequency of just over 1 Hz (exact timings dependent on the frame rate of the monitor), i.e. each grating alternated with a blank field every 494 ms. On the component adaptor side the two gratings were always presented in anti-phase. On the compound adaptor side the two gratings comprising the contour adaptor were presented with a temporal asynchrony of 0°, 21.5°, 43°, 90°, and 180° of phase angle (0, 59 ms, 118 ms, 247 ms, or 494 ms).

Mean PSE values for all asynchronies for all three observers individually and across the group are shown in Fig. 4B. There was a decrease in the CAE with increased temporal asynchrony, however, the decrease was gradual and for two observers there was still a significant adaptation effect with a 90° offset, only dropping to zero (equal adaptation in component and compound fields) when the components in the compound adaptor were completely out of phase (the two adaptor sides were identical). Unlike in the plaid adaptation experiment there was no significant reduction in magnitude of the CAE until the asynchrony was 90°.

## 4 Experiment 2. Integration of asynchronous components

Experiment 1 demonstrated that the temporal synchrony was important for the plaid aftereffect but less important for the CAE. However, both aftereffects were significantly greater than zero even with large asynchronies between the components comprising the compound, provided that some temporal overlap remained.

Experiment 2 aimed to measure the temporal window in which asynchronous stimuli can be integrated. It examined whether plaid and curvature aftereffects could be generated by stimuli where the components comprising the compound were presented in alternation and varied the temporal frequency of the component alternations to estimate the duration of the window in which integration can occur. In the limit, with very rapid alternation of the components, the stimulus approaches the original plaid stimulus. This adapting stimulus will still be referred to as the ‘compound’ adaptor although, in this experiment, the two components were never presented simultaneously.

Fig. 2B illustrates the presentation sequence for the components comprising the compound adaptor. The component adaptor gratings were always alternated at a rate of 1 Hz, while the compound adaptor gratings could alternate at one of seven frequencies between 1 Hz (equivalent to the component adaptor) and 42.5 Hz.

### 4.1 Plaid adaptation

The mean magnitudes of plaid adaptation for different temporal frequencies are shown in Fig. 5A. The overall trend was similar for all three participants. Unsurprisingly we see no plaid specific adaptation (95% confidence intervals include 0) at 1 Hz as the ‘compound’ and component adapting fields are identical at this frequency. Significant differences in adaptation between the compound and component fields were evident for temporal frequencies between 2 and 21 Hz. The effect peaked for alternation rates between 8.5 Hz and 14 Hz (a period of 71–118 ms), which is in agreement with the point at which Holcombe (2001) reported that two alternating gratings begin to be perceived as simultaneous rather than successive. These results add weight to the claim that gratings are processed independently before ‘being combined for awareness’ by a process that integrates information over a period of around 100 ms (Holcombe, 2001).

Two other notable trends are present in the data. First, the overall magnitude of the adaptation effects in this experiment were below 1 db, substantially lower than the 2.6 db effects seen for synchronous stimuli, and more in line with the adaptation magnitudes found for the asynchronous presentations in Experiment 1. This may indicate two underlying mechanisms for plaid-selective contrast adaptation; one sensitive to the temporal synchrony and the other less so. Second, at high frequencies the adaptation effect was eliminated. One potential explanation for this somewhat surprising result is that perceived contrast of the plaid adaptor, is reduced at high frequencies, resulting in weaker adaptation, relative to the component adaptor gratings.

### 4.2 Contour adaptation

The mean adaptation effects for the CAE at different temporal frequencies are shown in Fig. 5B. As the temporal frequency of the components comprising the compound adaptor increased, the magnitude of the CAE also increased. All observers showed significant selective adaptation with a temporal frequency of 4 Hz suggesting that components can be integrated without the components needing to be presented in synchrony. The frequency generating the largest CAE varied across observers, from 21 Hz to 42 Hz. This would indicate a mechanism with an integration window with a width in the region 24–48 ms, somewhat shorter than that found above for plaids.

There are two further differences between the temporal dependencies of the curvature and plaid aftereffects. First, although two out of three observers showed some drop in the magnitude of the CAE with high frequency alternation, the actual magnitude of the effect remained high. Second, the peak effect for the CAE with asynchronous components was not any lower than that produced with synchronous components. This suggests that the mechanisms involved in binding both synchronous and asynchronous components of contours act over a similar temporal scale.

## 5 Experiment 3. Integration of synchronous components

Experiments 1 and 2 investigated the sensitivity of the binding mechanism to temporal synchrony within compound stimuli. Experiment 3 aimed to determine how quickly conjunctions can be processed and whether the mechanisms can integrate over multiple presentations of the stimuli. Contour integration in Gabor path paradigms has been shown to be rapid, with detection occurring as fast as 13 ms for unmasked stimuli, or between 50 and 400 ms for masked stimuli, depending on the degree of curvature (Beaudot & Mullen, 2001; Hess, Beaudot, & Mullen, 2001; May & Hess, 2007). Hess et al. (2001) reported that detection required similar critical durations regardless of whether the path was presented in single or multiple exposures, suggesting that the mechanism does not integrate across multiple presentations of the same stimulus.

In this experiment the component gratings were always presented in synchrony and alternated with a blank field at one of seven frequencies between 1 Hz (equivalent to the component adaptor) and 42.5 Hz. Fig. 2C illustrates the presentation sequence for the components comprising the compound adaptor. The component side adaptor was identical to that used in Experiment 2, alternating at 1 Hz. While the total presentation duration of each grating in each condition is the same, the individual exposure duration varied from 12 ms (42.5 Hz) to 500 ms (1 Hz). From this we can examine the minimum presentation duration required for selective adaptation to occur and whether it is maintained (or enhanced) over multiple presentations of the stimuli.

### 5.1 Plaid adaptation

**Fig. 6:**
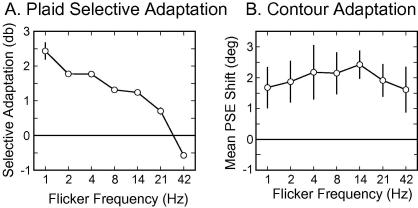
Group averaged magnitude of (A) the plaid adaptation effect (mean PSE shift in decibels) and (B) the CAE (mean PSE shift in degrees), at different flicker frequencies (Experiment 2). Error bars indicate the SEM across observers (plaids: *N* = 2; CAE: *N* = 3). 0 on the *y*-axis indicates the point of veridical equality.

Plaid-selective adaptation effects are plotted as a function of the flicker frequency in Fig. 6A. Adaptation was most apparent with long presentation times and gradually decreased as the flicker rate was increased from 1 Hz to 42.5 Hz. At 42.5 Hz the plaid adaptor no longer had any influence on subsequent probes, with adaptation becoming more apparent in the component-adapted field. These results may indicate that the shortest stimulus duration (12 ms) was not long enough for integration of the component gratings into a plaid and that the mechanism responsible for plaid adaptation was unable to compensate for this by integrating across separate presentations. The observed adaptation in the component-adapted field (negative values for selective adaptation) at high compound flicker rates could be explained by the reduction in perceived contrast of the plaid when flickered at 42 Hz. In this case both adapters would produce local component aftereffects but the apparent contrast in the component field would be higher and thus, produce the greatest effect.

### 5.2 Contour adaptation

The mean adaptation effects for different frequencies are shown in Fig. 6B. There was a large degree of variability in the responses of the three observers tested, but there was a general trend for the lowest magnitude CAE to be at the shortest presentation duration (greatest flicker frequency) and the peak magnitude to be at intermediate presentation durations. These results are broadly consistent with contour integration occurring at a minimum duration between 12 ms (42 Hz) and 25 ms (20 Hz). With presentations durations less than this two out of three observers showed little adaptation, suggesting that the initial integration could not be performed over multiple presentations.

The increase in CAE magnitude at intermediate flicker rates for most observers could indicate that sequential presentations, with individual durations long enough to allow binding, were integrated to some extent. This could increase the perceived duration of the contour presentation through temporal blurring, effectively increasing the adaptation duration for the contour. Alternatively, the increase at intermediate frequencies might be caused by greater attention to higher frequency stimuli, resulting in greater compound or local orientation adaptation to the higher frequency stimuli. However, if this were the case we might have expected to see a similar increase in plaid adaptation with intermediate flicker frequencies.

## 6 Discussion

The experiments described in this paper were designed to examine the temporal limits within which the visual system acts to bind two sinusoidal gratings into a compound pattern. We measured the magnitude of compound adaptation after varying the relative temporal phase, temporal frequency or flicker frequency of the components comprising the compound adaptor. Grouping was assumed to occur in cases where a compound adaptation effect was present, while disruption of either aftereffect was deemed to demonstrate segregation of the components. Our findings suggest that the CAE and plaid adaptation arise from different underlying neural mechanisms, with different temporal tuning properties. The method controls for adaptation to the component mechanisms (contrast adaptation to gratings and tilt aftereffects). The difference between these tuning properties should not, therefore, result from the underlying low-level mechanisms detecting contrast and orientation, even though these may also have different temporal tuning (c.f. Greenlee, Georgeson, Magnussen, & Harris, 1991; Magnussen & Johnsen, 1986).

### 6.1 Fast and slow grouping mechanisms

Previous work from our laboratory has shown that varying the spatial frequency content of the test probe or the components comprising the compound adaptor greatly diminishes compound adaptation (Hancock et al., 2010). The similarity between the spatial frequency tuning properties of plaid adaptation and the CAE led us to question whether both aftereffects might arise from a single, general binding mechanism. The current results argue against such an interpretation, however, with the two aftereffects showing very different temporal tuning characteristics. Most diagnostic of these differences is their respective sensitivity to temporal phase. Whereas very small temporal phase lags between the plaid components led to large decreases in the compound adaptation effect, the magnitude of the CAE was roughly proportional to the duration in which the contour components appeared together.

Plaid component segregation with small temporal phase lags is, however, in keeping with previous reports that temporal offsets as short as 10–40 ms could facilitate texture segmentation from a crowed background (Leonards & Singer, 1998; Leonards et al., 1996). Enhanced sensitivity for temporal phase has also been reported in overlapping patterns that were not part of a figure-ground display (Suzuki & Grabowecky, 2002), while Holcombe and Cavanagh (2001) demonstrated that observers could report colour and orientation combinations with a greater temporal resolution (∼30 Hz) when the stimuli were superimposed than when they were presented in adjacent locations (∼3 Hz). As in the aforementioned studies, temporal phase could represent an important cue for the segregation of plaid patterns into their components given that spatial cues are at a minimum when components overlap. On the other hand, when two components do not spatially overlap, as with contour stimuli, coding of temporal phase is less critical since spatial cues, such as good continuation and spatial phase alignment, can be utilised for grouping and segregation processes (Leonards et al., 1996). Indeed, Kiper et al. (1996) showed that contradictory or uninformative temporal information could be disregarded in cases where there was salient spatial information in figure-ground arrays.

Thus, it appears that there are at least two mechanisms involved in grouping and segmentation for plaids and contours; one that is highly sensitive to small differences in temporal phase, but is constrained by the spatial separation of the stimuli, and a second slower mechanism that is less constrained by spatial separation. Our data suggest that the former mechanism may contribute substantially to plaid-selective adaptation, while the latter may mediate the CAE. Similarly, previous studies have documented the need for two temporal processed to underlie segmentation (e.g. Forte et al., 1999). Rogers-Ramachandran and Ramachandran (1991, 1998) proposed the existence of a fast contour extracting mechanism that could use temporal phase changes to signal the presence of a contour without providing information about its polarity, and also a slow mechanism that signalled the surface qualities of the stimuli. Leonards and Singer (1998) similarly reported data supporting the notion of separate parallel mechanisms for processing textural and temporal cues. More recently, it has been suggested that it may be a basic principle of the visual system to divide most visual tasks into roughly-defined ‘fast’ and ‘slow’ temporal groups (Holcombe, 2009). According to this hypothesis, visual tasks that operate within a fine temporal limit occur at an early stage of processing, leading to almost instantaneous detection of motion or luminance edges for example. Whatever visual attributes fail to be processed at this early stage are passed further downstream, where more sophisticated neural machinery operating within a coarser temporal limit carries out any additional processing. This would suggest that the mechanism responsible for the plaid adaptation effect is situated at a relatively early processing stage (such as V1 or V2), while those mediating the CAE are located at a later stage (such as V4). It is important to note, however, that although there may be two general categories of visual tasks, this does not imply that there are only two underlying mechanisms across all classes of visual task. Tasks with different spatial and temporal characteristics may require binding mechanisms with different reliance on temporal structure.

An alternative explanation for the differences in the temporal tuning of the two aftereffects is that they are dictated by one mechanism that imposes a different temporal binding rule depending on the stimulus composition. Thus, a single underlying mechanism could explain all the aforementioned results by adjusting its response to match the stimulus properties. We have reason to believe that this is unlikely to be the case. Whereas the basis of the plaid-selective adaptation effect lies in contrast adaptation, we have previously shown that adaptation to a high-contrast contour has no effect on a subsequently presented contour probe (Hancock & Peirce, 2008). We deem it unlikely that this difference between the two aftereffects can be resolved without inference to two separate mechanisms, although further study is warranted to confirm this notion.

### 6.2 Interactions between fast and slow mechanisms

The enhanced sensitivity for temporal phase in plaid adaptation, relative to the CAE, implies that the underlying mechanism operates within a fast temporal limit in which it uses small differences in phase to segregate the plaid into its components. However, following the initial abrupt decrease in plaid-selective adaptation with increasing phase offset, there remained a small, but significant, selective aftereffect for all the other (asynchronous) conditions. This suggests that a second, more sustained, mechanism may also be at work in plaid adaptation that signals the presence of a plaid pattern regardless of context.

This notion is supported by the results of Experiment 2 where the components comprising the plaid or contour adaptor were presented in anti-phase and the temporal frequency of the alternations was varied. While a similar pattern of results is observed for each aftereffect in this experiment, there are two notable exceptions. First, the peak magnitude of the plaid adaptation effect (seen at a frequency of around 14–21 Hz) is greatly reduced in this experiment compared to the peak magnitude with simultaneously presented components. This is not the case for the CAE. This may suggest that the plaid-selective adaptation effects seen in Experiments 1 and 2 are governed by two separate mechanisms: one segregation process which is very sensitive to modulations of temporal phase, and a second with coarse temporal sensitivity that integrates components over 100 ms or more. In the case of the CAE a single mechanism with a coarse temporal sensitivity appears to mediate the integration/segregation of components in both experiments.

Second, in the plaid experiments, adaptation falls sharply at the highest alternation frequency tested (42 Hz). This is contrary to our *a priori* prediction that the compound adaptation effect would grow with increasingly rapid alternation rates, peaking at the fastest rate where the components are most clearly integrated perceptually. A similar trend was also apparent in the CAE data for two out of three participants, but to a much lesser extent. One contributing factor to this abrupt decrease in adaptation may be that this fastest alternation rate was the only condition to exceed the flicker fusion rate, with the strong perception of two flickering gratings giving way to the percept of a plaid. The accompanying reduction in the perceived contrast of the adaptor is likely to lead to a corresponding decrease in adaptation. The CAE is less sensitive to the contrast of the stimuli (Hancock & Peirce, 2008) and thus may be less affected by this issue.

A similar result occurs in the final experiment where the plaid aftereffect, and not the CAE, shows low-pass tuning for flicker frequency. That is, when the components comprising the compound stimuli were presented synchronously and flickered at different frequencies, plaid adaptation was most apparent with long presentation times and gradually deteriorated as the presentation changed to shorter, more frequent presentations of the adaptor. By 42 Hz the plaid adaptor no longer had any impact on the subsequently presented probe, with adaptation to the plaid pattern most apparent in the component-adapted field. One implication of this drop off of the plaid aftereffect at high flicker frequencies is that plaid-selective mechanisms do not integrate over multiple, rapid presentations of the plaid stimulus. This is in agreement with similar results derived from contour integration experiments (Hess et al., 2001). In contrast, the CAE shows no clear preference for the flicker rate of the stimulus. The reason for this difference between the two aftereffects remains unclear and warrants further study.

### 6.3 Sensitivity to subthreshold temporal manipulation

The enhanced sensitivity for temporal phase in plaid patterns extends to conditions where observers were not consciously aware of the stimulus onset asynchrony (phase offsets as short as 47 ms). In a control experiment, which adopted the same conditions as Experiment 1, observers required 98 ms (SEM = ±5) between the components to reliably detect that they were separated in time. While this figure is considerably larger than previously reported simultaneity thresholds of 55 ms (Elliott, Shi, & Surer, 2007; Elliott et al., 2006; VanRullen & Koch, 2003), the discrepancy between the measures is likely due to differences in the stimulus eccentricities used in the respective studies. This data adds to mounting evidence that subthreshold temporal modulations of stimuli can directly influence temporal judgments (e.g. Elliott et al., 2007; Parton, Donner, Donnelly, & Usher, 2006) and implies the role of an underlying mechanism that is highly sensitive to stimulus transients. It suggests that conscious awareness of temporal phase alignment is not a prerequisite for the use of this information, by some neural mechanisms, in segregating a plaid into its constituent components. Furthermore, it may indicate that the neural mechanism responsible for plaid detection (which we have characterized elsewhere as the result of an AND gate operation that sums two V1 outputs, see McGovern & Peirce, 2010; Peirce, 2007a, 2007b) exploits the synchronous/asynchronous nature of the low-level inputs. Such a mechanism would be very sensitive to temporal synchrony and would provide a parsimonious solution for determining whether two components belong to the same texture. This idea bears a strong resemblance to the temporal correlation hypothesis (Gray & Singer, 1989; Singer & Gray, 1995), which suggests that coherent neural oscillations could constitute a general mechanism underlying perceptual grouping. Under this framework, the low-level responses for each component converge on a common binding unit in the next-higher processing stage employing a “labelled line coding” principle, whereby a given combination of inputs always signals the same conjunction (Singer, 1999). However, this idea remains controversial (Shadlen & Movshon, 1999), and we, like others, (Dakin & Bex, 2002) believe it is impossible to verify using standard psychophysical procedures. Irrespective of how this procedure is implemented at cortical level, our results suggest that temporal-phase information plays an important role in the parsing of overlapping patterns.

In summary, the results of the experiments presented here indicate that the plaid adaptation effect and the CAE arise from mechanisms with differing temporal characteristics. The data might be explained by the existence of two binding mechanisms; a fast mechanism with high sensitivity to temporal synchrony (a resolution of around 20 Hz) and a separate mechanism with much coarser temporal sensitivity (around 2–4 Hz). We suggest that the former mechanism is responsible for plaid binding when the components are in synchrony or presented at frequencies above 21 Hz, and may be specialized for overlapping stimuli where there are few spatial cues. The latter mechanism is responsible for binding plaid and contour components across time and is likely to be sensitive to spatial cues such as good continuation.
